# Pericytes, Mesenchymal Stem Cells and the Wound Healing Process

**DOI:** 10.3390/cells2030621

**Published:** 2013-09-16

**Authors:** Stuart J. Mills, Allison J. Cowin, Pritinder Kaur

**Affiliations:** 1 Regenerative Medicine, Mawson Institute, Mawson Lakes, University of South Australia, South Australia 5095, Australia; E-Mail: stuart.mills@unisa.edu.au (S.J.M.); allison.cowin@unisa.edu.au (A.J.C.); 2 Epithelial Stem Cell Biology Laboratory, Research Division, Peter MacCallum Cancer Centre, St Andrew’s Place, Melbourne, Victoria 3002, Australia; 3 Sir Peter MacCallum Department of Oncology, University of Melbourne, Melbourne, Victoria 3002, Australia; 4 Department of Anatomy and Neuroscience, University of Melbourne, Melbourne, Victoria 3010, Australia

**Keywords:** wound healing, pericytes, skin, MSC

## Abstract

Pericytes are cells that reside on the wall of the blood vessels and their primary function is to maintain the vessel integrity. Recently, it has been realized that pericytes have a much greater role than just the maintenance of vessel integrity essential for the development and formation of a vascular network. Pericytes also have stem cell-like properties and are seemingly able to differentiate into adipocytes, chondrocytes, osteoblasts and granulocytes, leading them to be identified as mesenchymal stem cells (MSCs). More recently it has been suggested that pericytes play a key role in wound healing, whereas the beneficial effects of MSCs in accelerating the wound healing response has been recognized for some time. In this review, we collate the most recent data on pericytes, particularly their role in vessel formation and how they can affect the wound healing process.

## 1. Pericyte Morphology

**Figure 1:**
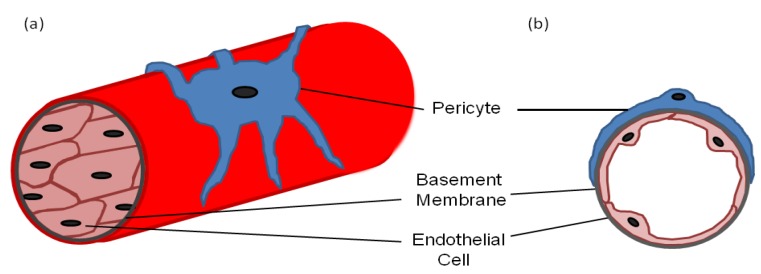
(**a**) A diagram of a longitudinal section of a blood vessel covered by a pericyte; (**b**) A cross-sectional diagram of a blood vessel showing the endothelial cell and pericyte interaction with a common basement membrane.

Pericytes—also known as mural cells, Rouget cells, ito cells in the liver, mesangial cells in the kidney and adventitial reticular cells in the bone marrow—are found throughout the body in a variety of forms [1,2]. Most pericytes arise from the mesoderm, such as those found around the vessels in the trunk of the body, whereas others can arise from the neural crest, such as brain pericytes [3,4]. Pericytes can even arise directly from endothelial cells and the bone marrow [5,6]. However, universally pericytes are situated on the outer surface of blood capillaries where they interact with the underlying endothelial cells (Figure 1) and are often covered in the same basement membrane [7]. They are fibroblast-like in appearance but have several long processes with a prominent nucleus and little cytoplasm. The processes are usually in contact with more than one endothelial cell via adhesion plaques and peg and socket contacts, which allow direct contact between the two cell types [8,9,10,11]. The adhesion plaques, as the name suggests, adhere the pericytes to the endothelial cells, whereas the peg and socket contacts also allow diffusion of ions and molecules between the cytoplasm of the two cell types [10]. The adhesion plaques maintain their contact via fibronectin deposits, whereas the peg and socket contacts include tight-, gap- and adherence junctions containing N-cadherin and β-catenin [12,13]. 

Three types of pericytes are thought to exist depending on their position on the blood vessel. These include: pre-capillary, mid/true-capillary and post-capillary pericytes [14]. Mid-capillary pericytes differ from the other two in that they lack α-smooth muscle actin within the cell and are elongated and more spindle shaped [15]. The pre- and post-capillary pericytes are shorter, more stellate in shape, and contain varying amounts of α-smooth muscle actin [15]. The ratio of pericytes to endothelial cells fluctuates in different tissues—thus in the retina and central nervous system the ratio is 1:1; in the skin and lung the ratio is 1:10; and for tissues such as striated muscle the ratio can be as little as 1:100 [16]. This variation in number is thought to be linked to the function of the tissue in which the vessel resides *i.e.*, the greater the blood pressure within the vessel the higher the number of pericytes and therefore the greater the degree of control of that vessel [16]. This may explain why more pericytes can be identified on larger vessels, and why they are also more abundant in the distal legs and feet giving support to the hypothesis that the greater the hydrostatic pressure of the vessel the greater the number of pericytes [17].

## 2. Markers for Isolating/Identifying Pericytes

Early investigations into pericytes involved micro-dissection of tissues to remove the vessel and subsequent culture of the endothelial cells and pericytes. The pericytes obtained out-competed the endothelial cells *in vitro* to leave a “pure culture” of pericytes [16]. Studies to identify better markers for pericytes have had limited success. Initially, methods to identify pericytes from other dermal cells such as fibroblasts, endothelial and smooth muscle cells, relied on immuno-histochemistry for a combination of cytoskeletal proteins thought to be uniquely expressed by pericytes [18]. Herman and D’Amore [19] discovered that pericytes could be distinguished from smooth muscle cells and endothelial cells by their unique expression of muscle and non-muscle isoactins. Smooth muscle cells were found to strongly express muscle isoactin but had diminished levels of non-muscle isoactins. Conversely, endothelial cells stained strongly for non-muscle isoactins but not for muscle isoactin. Pericytes, however, were found to stain for both muscle and non-muscle isoactin. Initial searches for a single marker of pericytes utilized an antibody labeled 3G5 to identify retinal pericytes, but was subsequently found to also stain endothelial cells from other tissues [16]. A surface differentiation antigen Thy 1.1 has also been put forward as a pericyte marker—but it was also present on thymus derived-lymphocytes and astrocytes [18]. 

More recently pericytes have been shown to express a range of receptors and proteins including platelet derived growth factor receptor-β (PDGFR-β), epidermal growth factor receptor (EGFR), adenosine A_2_ receptors, α-smooth muscle actin (αSMA), desmin, NG2 proteoglycan, aminopeptidase A and N, and regulator of G-protein signaling 5 (RGS5). [12,20,21,22]. Although these markers are routinely used in the identification of pericytes, none is completely specific for pericytes and neither are they expressed by pericytes in all tissues and organs. The problem could arise from plasticity of pericytes, which can express different markers within different tissues and at different times of development [13]. For example, RGS5 is expressed on activated pericytes during tumor development and vascular remodeling but not at other times. The reasons for the multiple phenotypes may be due to the various origins of the pericytes. While most arise from the mesoderm, others can arise from the neural crest [3,4]. Thus, the lack of a single marker for pericytes can give rise to misinterpretation of results and defining the true role of pericytes becomes fraught with difficulties.

## 3. Pericytes and the Formation of Blood Vessels

Blood vessels are formed early on during embryogenesis from the mesoderm in a process known as vasculogenesis [23]. Initially hemangioblasts form into blood islands, which consist of endothelium and primitive blood cells [24]. These form into tube-like structures in response to TGF-β, which then branch and remodel, during the process of angiogenesis, forming the early vascular network [16,23]. Angioblasts, progenitors of the hemangioblasts, have been found to make up the majority of the endothelial cells of the major vessels in the trunk and limbs and their migration is in response to VEGF [25,26]. When the endothelial cells invade, they recruit the primordial local mesenchymal stem cells to the vessel and aid in their differentiation into mural cells such as pericytes and smooth muscle cells [24]. It has been reported that pericytes can at this point inhibit endothelial cell proliferation and promote their differentiation via expression of TGF-β [27,28,29]. Interestingly, Hirschi *et al.* [30] have also shown that the endothelial cells themselves can inhibit pericyte proliferation via PDGF-B, independently of TGF-β expression, where there is an absence of cell-cell contact. When cell-cell contact is permitted proliferation of both cell types was shown to be inhibited. Both cells are then thought to contribute to the formation of the basement membrane [7,31]. Transforming growth factor-β1 (TGF-β1) is required for this initial formation of the blood vessels because when depleted or when genes encoding their formation are knocked-out such as activin-receptor like kinase 1 (*alk1*), endoglin and TGF-β receptor II (TGFR-βII), this results in cardiovascular defects, which are embryonic lethal [32,33,34]. TGF-β signaling through ALK1/Smad1/5 is thought to promote proliferation and ALK5/Smad2/3 to promote differentiation of endothelial cells [35]. The release of TGF-β through ALK5 signaling in endothelial cells has also been reported to result in the differentiation of pericytes to smooth muscle cells [36]. Interestingly smooth muscle cells and pericytes both readily express latent TGF-β1, which then becomes activated once there is contact with the endothelial cell resulting in the expression of α-smooth muscle actin [37,38]. In other reports, this contact was not essential and growth inhibition of the endothelial cells could still be observed [39,40].

PDGF and the PDGFR-β are both expressed by endothelial cells during early human development and may promote their proliferation in an autocrine manner in these cells [41]. Mural cells exhibit continuous expression of the PDGFR-β, which is essential for their recruitment to the vessel wall. *In vitro* experiments support this hypothesis—addition of neutralizing antibody to PDGF inhibits smooth muscle cell migration towards PDGF-BB expressing endothelial cells via down-regulation of the sphingosine-1-phosphate pathway (S1PR1/S1PR3) and an induction of haem oxygenase-1 (HO-1) expression [1,42,43]. Interestingly PDGF knockout mice were found to be embryonically lethal. In these embryos mural cells were not recruited to the vasculature and they died from hemorrhage, suggesting that the expression of PDGF is essential for fetal development [41]. 

Basic fibroblast growth factor (bFGF) is also expressed by endothelial cells and is chemotactic for smooth muscle cells [44]. Another growth factor involved in vessel formation is heparin binding-epidermal growth factor (HB–EGF), which promotes smooth muscle cell and pericyte proliferation [1]. These studies suggest that mural cell proliferation and migration are regulated by endothelial cells and vice-versa; thus, both populations of cells have interdependent regulatory mechanisms [1]. 

## 4. Pericytes and the Regulation and Maintenance of Blood Vessels

Some of the main functions of pericytes are to regulate blood flow and regulate the perfusion of fluids, cells and proteins between the capillaries and the surrounding tissue [16]. The high levels of α-smooth muscle actin and myosin present in pericytes suggests that these cells can regulate blood flow and the pressure within the vessel that they are attached to in a similar manner to the smooth muscle cells of the larger vessels [45]. These cells tend to localize to a position either side of the mid-capillary bed. *In vitro* experiments carried out by Das *et al.* [46] confirmed the contractile properties of pericytes—thus permeabilizing the cells and administering ATP led to contraction of the pericytes, but not in endothelial cells or epithelial cells under the same conditions. Pericytes are also able to contract collagen lattices and silicon substrates, and the contraction of pericytes and vessel constriction appears to be initiated by an up-regulation of endothelin-1 and by down-regulation of iNOS production from the endothelial cells [40,47]. In contrast, substances such as cAMP and cGMP promote pericyte relaxation [48]. Joyce *et al.* [49] demonstrated that cyclic GMP-dependent protein kinase within pericytes was expressed in a similar manner to smooth muscle cells. While this data supports the idea that the contraction of pericytes could protect the vessel from excessive stretching during processes such as inflammation and control for local pressure in the vessel by contracting down the cross-sectional area of that vessel, it remains difficult to ascertain to what extent this occurs *in vivo* in microvessels. The above studies show that there are similarities between pericytes and smooth muscle cells and that perhaps the two cell types represent a continuum rather than existing as two distinct phenotypes.

There is a population of pericytes distinct from smooth muscle cells, which express no α-smooth muscle actin in the mid-capillary. These pericytes are situated at endothelial cell junctions and are thought to open these junctions to allow the perfusion of fluids, cells and proteins through the capillary wall rather than play a role in the control of blood flow [1,16]. In these areas between the pericytes there is a reduction in the concentrations of key vascular basement membrane proteins including laminin-10, collagen IV and nidogen-2 and Wang *et al.* [50] have shown that these areas are utilized by neutrophils to enter the perivascular space. The discovery of these areas of low concentration basement membrane proteins provides a plausible mechanism for the extravasation of blood cells without causing major disruption to the vessel integrity and promoting a local healing response.

In adults, when existing blood vessels are damaged through injury and new vessel formation is required, pericytes already present on the vessel wall detach and the vascular basement membrane degraded. This removes the inhibitory influence of the pericytes on endothelial cell proliferation allowing endothelial cells to begin to divide and migrate into the surrounding matrix to form new vessels [51,52]. This proliferation and migration may be influenced by endothelial cell integrin-matrix interactions [53]. The provisional matrix formed after injury contains both fibronectin and vitronectin, which have been found to promote endothelial cell adhesion and migration [54]. In particular the expression of integrin receptors for fibrin/fibronectin, αvβ3, has been shown to increase, as well as the rate of angiogenesis, when endothelial cells were plated on fibronectin compared to when they were plated on collagen. Once primitive vessels are formed, the pericytes are then recruited back by PDGF-B expressed by the endothelial cells. Once situated back on the vessel wall, the pericytes promote endothelial cell differentiation and inhibit proliferation leading to vessel stability [55].

## 5. Mesenchymal Stem Cell Properties of Pericytes

Early studies with pericytes hinted that they may play a greater role than just maintaining the vasculature—an increasing body of evidence has since identified them as mesenchymal stem cells (MSCs). To date pericytes have been shown to differentiate into chondrocytes, adipocytes, osteoblasts, phagocytes and granulocytes [1,56,57,58]. These studies strongly suggest that pericytes are indeed mesenchymal stem cells capable of giving rise to other types of mesenchymal cells. Interestingly this differentiation fate appears to be highly dependent on neighboring cells. Moreover, it has also been suggested that all MSCs are pericytes [59].

Early studies by Kristensson and Olsson [60] showed that systemic injections of protein tracers in mice led to an accumulation of the tracers in the pericytes of the brain and spinal cord. This suggested that pericytes could absorb molecules by pinocytosis in a similar manner to macrophages. This was confirmed in a study where they were shown to clean soluble molecules from the extracellular fluid in the blood brain barrier [61]. To further support the hypothesis that pericytes can act as phagocytes, pericytes have since been shown to express similar properties such as scavenging and Fc receptors and to even have phagocytic activity [61]. They have also been shown to migrate to and become incorporated in bone tissue as osteoblasts lending support to the idea that they have stem cell-like properties and can convert into multiple cell types [62]. 

More recent studies by Farrington-Rock *et al.* [57] investigated the differentiation potential of pericytes into chondrocytes. They found that when placed in chondrogenic medium, pericytes expressed the chondrocyte markers Sox-9, aggrecan and type II collagen. Under these conditions the pericytes formed into well-defined pellets embedded in an extracellular matrix containing high levels of sulfate proteoglycans and collagen II. In contrast, endothelial cells grown in the same conditions did not display chondrogenic differentiation. Kirton *et al.* [63] have shown that TGF-β3 initiates Wnt/β-catenin signaling leading to the differentiation of pericytes into chondrocytes in pellet cultures. When this signaling was blocked using a recombinant adenovirus encoding the dominant negative T-cell factor-4 (RAd/dnTCF), chondrogenesis was inhibited and there was a decline in collagen II and Sox-9 expression. More recent work from our own laboratory has confirmed that skin dermal pericytes can also form chondrocytes in pellet cultures, in addition to undergoing adipogenesis or osteogenesis under the appropriate inductive conditions *in vitro* [56].

Early investigations showed that capillary pericytes could convert to immature adipocytes in response to thermal injury [58]. More recently, pericytes placed in adipogenic medium alternatively expressed peroxisome proliferator-activated receptor-γ2 (an adipocyte transcription factor) and the cells contained lipid droplets. This was also shown *in vivo* with pericytes but not endothelial cells [57]. Pericytes can also convert to smooth muscle cells and interestingly the reverse has also been observed —that is smooth muscle cells can convert to pericytes, further supporting the hypothesis that these cells have stem cell-like properties [64,65].

## 6. Mesenchymal Stem Cells and Wound Healing

Since the discovery of MSCs and the creation of stable cell lines, investigations into their application have increased hugely. Interestingly, MSC circulation in peripheral blood increases with injury and depends on the size of the injury [66]. Most investigations into the application of MSCs to wounds have a positive effect even if the outcomes have been varied; whether this is due to the cells applied or the wounding model used remains to be elucidated. 

One of the earlier studies by Nakagawa *et al.* [67] implanted porcine skin equivalents into wounds made on the dorsal skin of nude mice. The implants either contained human MSCs (hMSCs) or a combination of hMSCs and bFGF. Both treatments accelerated wound closure after 7 days; although these authors claimed that the hMSCs converted to keratinocytes within the wound, the data presented were at best equivocal. Bone marrow derived MSCs (BM-MSCs) also contribute to wound healing in skin. Wu *et al.* [68] used an excisional wound splint model in mice and injected GFP labeled BM-MSCs around the wound margins. This led to an increased rate of wound closure, re-epithelialization and angiogenesis. These authors also claimed that the BM-MSCs contributed to the re-growth of the epidermis and production of keratins, but a close examination of the data reveals that a significant number of GFP^+^ BM-MSCs cells were found associated with dermal appendages, rather than the epidermis. Given the absence of convincing flow cytometric data exhibiting dual staining for keratins and GFP, and the surprisingly high number of GFP^+^cells (almost two million) in the sham-operated mice, it is difficult to conclude that BM-MSCs contribute to the regenerated epithelium, particularly in the interfollicular epidermis. Wu *et al.*, did however convincingly demonstrate increased angiogenesis in the presence of BM-MSCs, and further that BM-MSC conditioned media contained higher levels of VEGF and angiopoietin-1 compared to controls which demonstrably promoted endothelial cell tube formation [68]. 

Further evidence for the notion that BM-MSCs can improve skin wound healing is widely available in the literature—Falanga *et al.* [69] showed that human BM-MSCs could be combined with either fibrinogen or thrombin and applied to chronic wounds in the form of a spray, which formed a gel over the wound containing the MSCs. This application led to a reduction in size of the chronic wound and the rate of healing increased, correlating with the number of cells applied. Similarly, the application of MSCs in fibrin to elliptical full thickness wounds in diabetic mice, also led to accelerated wound healing. These findings [69] were supported by the work of Badillo *et al.* [70] who investigated the effects of fetal liver MSCs in wound healing in diabetic mice. Badillo *et al*. [70] made 8mm punch biopsy full-thickness wounds in the dorsal skin of mice and treated them with GFP-labeled MSCs. The results correlated with the data from the Falanga *et al.* [69] study, showing an increase in the rate of healing with a smaller dermal gape and increased contraction when compared to the control wounds. An increase in matrix formation and vessel formation was also reported and could probably be attributed to the observed up-regulation of growth factors including EGF, TGF-β1 and stromal-derived growth factor-1α. Notably, Badillo *et al.*, reported that the majority of the GFP labeling was restricted to myofibroblasts within the dermis rather than keratinocytes in the epidermis in direct contrast to the claims of Nakagawa et al [67] and Wu et al [68]. Interestingly, MSCs also appear to influence the inflammatory response as an up-regulation of MIP-1α and β has been reported with an increase in the number of macrophages infiltrating the wound [71].

Thus, an increase in the rate of healing has been observed in several animal wounding models after treatment with MSCs and notably, many of these studies use BM-MSCs. However, adipose tissue and umbilical cord- derived MSCs can also improve wound repair [72,73]. After treatment with MSCs there is often an increase in the rate of re-epithelialization, an increase in dermal matrix production and an up-regulation of angiogenesis. It appears that MSCs promote wound healing by supplying the required cytokines and growth factors and by differentiating into different cell types within the wound including endothelial cells, monocytes and pericytes [68,74,75]. It has been claimed that the application of MSCs to wounds not only improves and accelerates the wound healing response but can even promote the formation of new skin appendages, such as sweat glands, which often fail to reform after injury [76]. If this can be proven beyond doubt by further investigation, the therapeutic potential of MSCs from a number of tissues will be immense. 

## 7. Pericytes and Wound Healing

The wide and successful use of BM-MSCs in wound healing models suggests that there may be great potential for these cells in treatments for burns and accidental injuries to the skin. However, these cells are often difficult and painful to isolate for human use and therefore a more easily accessible and plentiful source is required. Pericytes, as MSCs, could be an ideal source—however their role in wound healing is still poorly understood and this may be due in part to the lack of a single marker to identify these cells. 

It has been known for some time that mid- and post-capillary venules are rich in pericytes. In these blood vessels, pericytes are thought to open inter-endothelial junctions to allow protein leakage and cell migration towards the wound site [17]. Indeed pericytes have been found to relocate to the endothelial cell junctions and to even form umbrella-like structures over these junctions preventing the release of proteins and cells, instead retaining them within the vessel wall [77,78]. This suggests that they may play a role in the inflammatory process controlling the rate at which inflammatory cells leave the blood stream and migrate to the wound site. Pericytes may also play a role in the matrix formation process of wound healing. For instance, bovine retinal pericytes produce the main constituents of the extracellular matrix including: fibronectin, laminin, fibronectin, thrombospondin, collagen I and collagen III [16,79,80]. 

PDGF-B appears to play a role in pericyte and fibroblast recruitment to the wound site. When imatinib (a PDGF blocker) was applied to 4mm excisional wounds on the dorsum of mice, the rate of wound closure was inhibited and there was a decrease in cell proliferation [81]. A reduction in myofibroblast numbers within the wound was also reported, and although their differentiation was not inhibited, fibroblast proliferation and migration was. Imatinib treatment also led to collagen I production being limited to the wound margins and reduced along with fibronectin expression. Pericytes are widely reported to express PDGF-β receptor and can leave the blood vessel to migrate into the wound site. Rajkumar *et al.* [81], reported inhibition of pericyte migration and proliferation upon blockade of the PDGF-β receptor with imatinib. However, it is worth noting that although pericyte function can be inhibited by imatinib, its effects are not pericyte-specific and as such, the observed effects on wound healing may not be solely due to the lack of functional pericytes.

One of the few studies to investigate the role of pericytes in wound healing reported by Sundberg and colleagues has suggested that despite their inherent MSC properties, pericytes may not actually be beneficial for the wound healing process [82]. In this study wounds were stained using prolyl-4-hydroxylase beta subunit (P-4-H) to identify collagen production and high molecular weight-melanoma associated protein (HMM-MAA), integrin α5, SMA and PDGFR-β to identify pericytes. Sundberg *et al.* found that the population of pericytes leaving the blood vessels and migrating into the wounds were lacking SMA expression but were high in collagen expression. These researchers suggested that pericytes were not converting into myofibroblasts and aiding contraction, but instead were producing collagen and promoting fibrosis [82]. In a similar study investigating the role of pericytes in burn wounds, Popescu *et al.* [83] also suggest that pericytes were not differentiating into myofibroblasts, but in fact, the resident fibroblasts were.

## 8. Discussion

The above studies demonstrate that pericytes have the potential to have a wider role than just promoting vessel growth and stability. As interest in this cell type has increased, they have been shown to be essential in the formation of the vascular network and in development. Pericytes also appear to have many stem cell-like properties and can differentiate into adipocytes, chondrocytes, phagocytes and osteoblasts leading to their classification as MSCs. This has even led to suggestions that all MSCs are pericytes and that the vessels act as a niche for these MSCs. There does however seem to be a distinction between pericytes and MSCs derived from other sources in particular BM-MSCs. This distinction appears more obvious when investigating their effects on the wound healing process. In studies that applied BM-MSCs to wounds, the majority found beneficial effects, *i.e.*, accelerated wound healing. This has not been the case with pericytes with reports thus far indicating that they were more likely to promote fibrosis rather than accelerate wound healing. Overall this suggests that pericytes may maintain a degree of plasticity in their ability to differentiate to other cell types - but that this plasticity may actually be more limited and may affect their inherent function when compared to BM-MSC.
